# Arginase Activity - A Marker of Disease Status in Patients with Visceral Leishmaniasis in Ethiopia

**DOI:** 10.1371/journal.pntd.0002134

**Published:** 2013-03-28

**Authors:** Tamrat Abebe, Yegnasew Takele, Teklu Weldegebreal, Tom Cloke, Ellen Closs, Camille Corset, Asrat Hailu, Workagegnehu Hailu, Yifru Sisay, Karina Corware, Margaux Corset, Manuel Modolell, Markus Munder, Fabienne Tacchini-Cottier, Ingrid Müller, Pascale Kropf

**Affiliations:** 1 Department of Microbiology, Immunology and Parasitology, Addis Ababa University, Addis Ababa, Ethiopia; 2 Department of Biochemistry, WHO Immunology Research and Training Center, University of Lausanne, Lausanne, Switzerland; 3 Gondar University Leishmaniasis Research and Treatment Centre, Gondar University, Gondar, Ethiopia; 4 Department of Medicine, Section of Immunology, Imperial College London, London, United Kingdom; 5 Institute of Pharmacology, University Medical Center of the Johannes Gutenberg University Mainz, Mainz, Germany; 6 College of Medicine and Health Sciences, University of Gondar, Gondar, Ethiopia; 7 Immunology and Infection Department, London School of Hygiene and Tropical Medicine, London, United Kingdom; 8 Department of Cellular Immunology, Max-Planck-Institute for Immunobiology and Epigenetics, Freiburg, Germany; 9 Third Department of Medicine (Hematology, Oncology, and Pneumology), University Medical Center Mainz, Mainz, Germany; National Institute of Health, United States of America

## Abstract

The underlying mechanisms resulting in the profound immune suppression characteristic of human visceral leishmaniasis (VL) are not fully understood.

Here, we tested the hypothesis that arginase, an enzyme associated with immunosuppression, is higher in patients with VL and contributes to impaired T cell responses. We recruited patients with VL before and after treatment and healthy controls and measured the arginase metabolism in the blood of these individuals. Our results show that arginase activity is significantly higher in the blood of patients with active VL as compared to controls. These high levels of arginase decline considerably once the patients are successfully treated. We identified the phenotype of arginase-expressing cells among PBMCs as neutrophils and show that their frequency was increased in PBMCs of patients before treatment; this coincides with reduced levels of L-arginine in the plasma and decreased expression levels of CD3ζ in T cells.

## Author Summary

Leishmaniases, a group of diseases caused by a parasite, Leishmania, belong to the most neglected tropical diseases: they are mainly found in low-income countries and affect the poorest populations. These parasites infect cells of the immune system called macrophages, which can kill the intracellular parasites when they receive the right signals from other cells of the immune system, the lymphocytes. During the active phase of visceral leishmaniasis, it has been shown that lymphocytes lose their capacity to instruct the macrophages to kill the intracellular parasites. Here, we show that the levels of an enzyme, arginase, are significantly increased in the blood of patients with visceral leishmaniasis, but decrease to the same levels as those of healthy controls following successful treatment. Arginase has the capacity to deplete an amino acid, L-arginine, which is crucial for the activation of lymphocytes. Indeed, our results show that the levels of this amino acid are considerably decreased in patients with visceral leishmaniasis. Our results suggest that during the active phase of visceral leishmaniasis, increased arginase results in the depletion of L-arginine, which is responsible for the incapacity of lymphocytes to send the adequate signals to the macrophages.

## Introduction

Visceral leishmaniasis (VL) is a neglected tropical disease caused by parasites of the *Leishmania (L.) donovani complex*. The incidence of VL is estimated to be 500 000 cases every year, with approximately 50 000 deaths, predominantly in India, Bangladesh, Brazil, Nepal and Sudan. It inflicts an immense toll on the developing world and impedes economic development; it is the second biggest parasitic killer in the world after malaria, with an estimated loss of 2.3 million disability-adjusted life years. There is no efficient vaccine; currently used chemotherapy is toxic and increasing drug resistance is reported [1]. VL can be asymptomatic or can manifest as a progressive disease characterised by hepatosplenomegaly, fever, weight loss, hyperglobulinemia and pancytopenia [1]. In Ethiopia, VL is caused by *L. donovani* and it is one of the most significant vector-borne diseases; Ethiopia has the second largest number of VL cases in sub-Saharan Africa with an estimated annual burden of 4500 to 5000 new cases [2]. Sodium stibogluconate (SSG) is still the main drug used as first line of treatment in Ethiopia [2] and a case fatality rate of 13% has been reported [3]. VL is worsened by malnutrition and HIV co-infection, and treatment access is often difficult because of the remote location of areas endemic for VL.

Based on extensive studies in mouse models, healing of disease and host protection against *Leishmania* infection is dependent on the development of a T helper (Th)1 type response, characterised by the production of IFN-γ and nonhealing is associated with an IL-4-dominated Th2 type response (reviewed in [4]). However, concentrations of these two cytokines in plasma of symptomatic patients with visceral leishmaniasis (VL patients) do not always correlate with clinical outcome: studies have reported both an increase [5], [6], [7], or decrease [9] in IFN-γ levels during the active phase of VL. Whereas IL-4 increases in plasma of VL patients in some studies [8], [9], [10], it has also been shown to be below detection limit in other cases [6], [11]. In contrast, increased levels of IL-10, a potent immunosuppressive cytokine, have consistently been associated with symptomatic VL (reviewed in [12]), suggesting that rather than a set Th1 or Th2 type response, IL-10 plays a crucial role in chronic nonhealing/or symptomatic VL. Indeed, one of the key immunological characteristics of active VL is a profound immunosuppression, as demonstrated by the failure of PBMCs to produce IFN-γ and proliferate in response to *Leishmania* antigen; this impaired capacity to respond to antigenic challenge is however restored following successful chemotherapy [6] and reviewed in [12], [13].

Arginase-induced L-arginine metabolism has been identified as a potent mechanism of immune suppression [14], [15], [16]. We have recently shown in an experimental model of cutaneous leishmaniasis that high arginase activity, a hallmark of nonhealing disease, is primarily expressed locally at the site of pathology. The high arginase activity caused local depletion of L-arginine, which impaired the capacity of CD4^+^ T cells in the lesion to proliferate and to produce interferon-gamma, while T cells in the local draining lymph nodes responded normally. Healing resulted in control of arginase activity and reversal of local immunosuppression. Furthermore, inhibition of arginase as well as supplementation with L-arginine restored T cell effector functions and reduced parasite growth at the site of lesions [17]. In addition, it has been recently shown in a hamster model of visceral leishmaniasis that increased arginase activity resulted in impaired control of infection [18].

In the present study, we tested the hypothesis that during human visceral leishmaniasis, arginase activity increases and results in depletion of L-arginine.

## Materials and Methods

### Subjects and sample collection

The study was approved by the Ethiopian National Research Ethics Review Committee (NRERC, reference 310/18/03), by Addis Ababa University Medical Faculty Institutional Review Board (IRB, reference 023/2009) and by the Joint UCL/UCLH Committees on the Ethics of Human Research (Committee Alpha, reference 09/H0715/93). For this study, 26 patients with visceral leishmaniasis were recruited from the Leishmaniasis Treatment and Research Center of Gondar University Hospital before treatment. The exclusion criteria were age (<5), tuberculosis, malaria, HIV and pregnancy; no women presented with visceral leishmaniasis during our study, all patients were male migrant workers. The diagnosis of VL was based on positive serology (rK39) and presence of amastigotes in spleen or bone marrow aspirates. Fourteen male healthy controls with no prior history of VL were recruited among the staff (10 controls) in the clinic and from the patients' household contacts (4 controls). Informed written consent was obtained from each patient or parent/guardian and control. 10–20 ml of blood was collected in EDTA tubes before the treatment started. All patients were treated with 20 mg/kg/day of SSG for 30 days and showed an initial clinical cure rate of 100% after treatment. In addition there was no significant treatment related adverse event.

A further 10 ml of blood was collected from 14 patients 21 to 28 days after treatment had started. Of note, in all the tests performed in this study, no significant differences were obtained between patients after 3 weeks of treatment as compared to 4 weeks of treatment.

It was not possible to always analyse the samples from all patients before and after treatment for two reasons: poor medical conditions of the patients at arrival in the hospital and/or frequent electricity cuts that did not allow for immediate processing of the blood.

Plasma was isolated by centrifuging 1 ml of blood at 1800 rpm for 10 min and was frozen at −20°C until further use. Peripheral blood mononuclear cells (PBMCs) were isolated by density gradient centrifugation on Histopaque-1077 (Sigma). Cells were counted by trypan blue exclusion, washed in phosphate buffered saline (PBS) and were used immediately for flow cytometry; PBMCs used for arginase and protein determination were immediately resuspended in lysis buffer (0.1% Triton X-100, 25 mM Tris-HCl and 10 mM MnCl_2_, Sigma) and frozen at −20°C until further use.

### Laboratory evaluation

The following tests were used to diagnose the presence of HIV: KHB Shanghai Kehua Bio-engineering Co. Ltd and Chembio HIV 1/2 STAT-PAK; Uni-Gold (Trinity Biotech PLC) was used to resolve ambiguous results.

CD4^+^ and CD8^+^ T cell counts were determined using a BD Multi TEST kit (BD Biosciences) and acquisition was performed using a FACSCalibur (BD Biosciences). Haematological data (platelet counts, white blood cell counts, hematocrit and haemoglobin) were obtained with a COULTER Ac•T diff Hematology Analyzer.

### Determination of arginase activity

The enzymatic activity of arginase in PBMCs and in plasma and protein concentration of PBMC samples was measured as previously described [19].

### HPLC quantification of L-arginine

L-arginine concentrations were determined in plasma obtained from citrated blood either directly or after concentrating cationic amino acids using Oasis MCX ion exchange columns (Waters, Eschborn, Germany) as described before [20].

### Flow cytometry

Antibodies used were as follows: anti-CD4 (clone 13B8.2, Beckman Coulter), anti-CD8 (clone RPA-T8, BD Biosciences), anti-CD3ζ (Santa Cruz: clone 6B10.2), anti-CD14 (BD Pharmingen: cloneM5E2), anti-CD15 (Clone H198, BD Pharmingen) and anti-CD63 (Beckman Coulter: CLBGran/12); anti-arginase I (HyCult Biotechnology: clone 6G3) and the isotype control (BD Pharmingen: clone MOPC21) were coupled with Alexa FluorR 488 (Molecular Probes). Cells were washed with PBS, the fixation step was performed with 2% formaldehyde in PBS and the permeabilisation step with 0.5% saponin in PBS.

The determination of intracellular arginase was performed as described in [21]. The percentages for the isotype controls were <1%. Acquisition was performed using a FACSCalibur (BD Biosciences) and data were analyzed using Summit v4.3 software.

### Statistical analysis

Data were evaluated for statistical differences using a two-tailed Mann-Whitney test (GraphPad Prism 5) and differences were considered statistically significant at *p*<0.05. Unless otherwise specified, results are expressed as median± SEM.

## Results

### Clinical data

**Figure 1:**
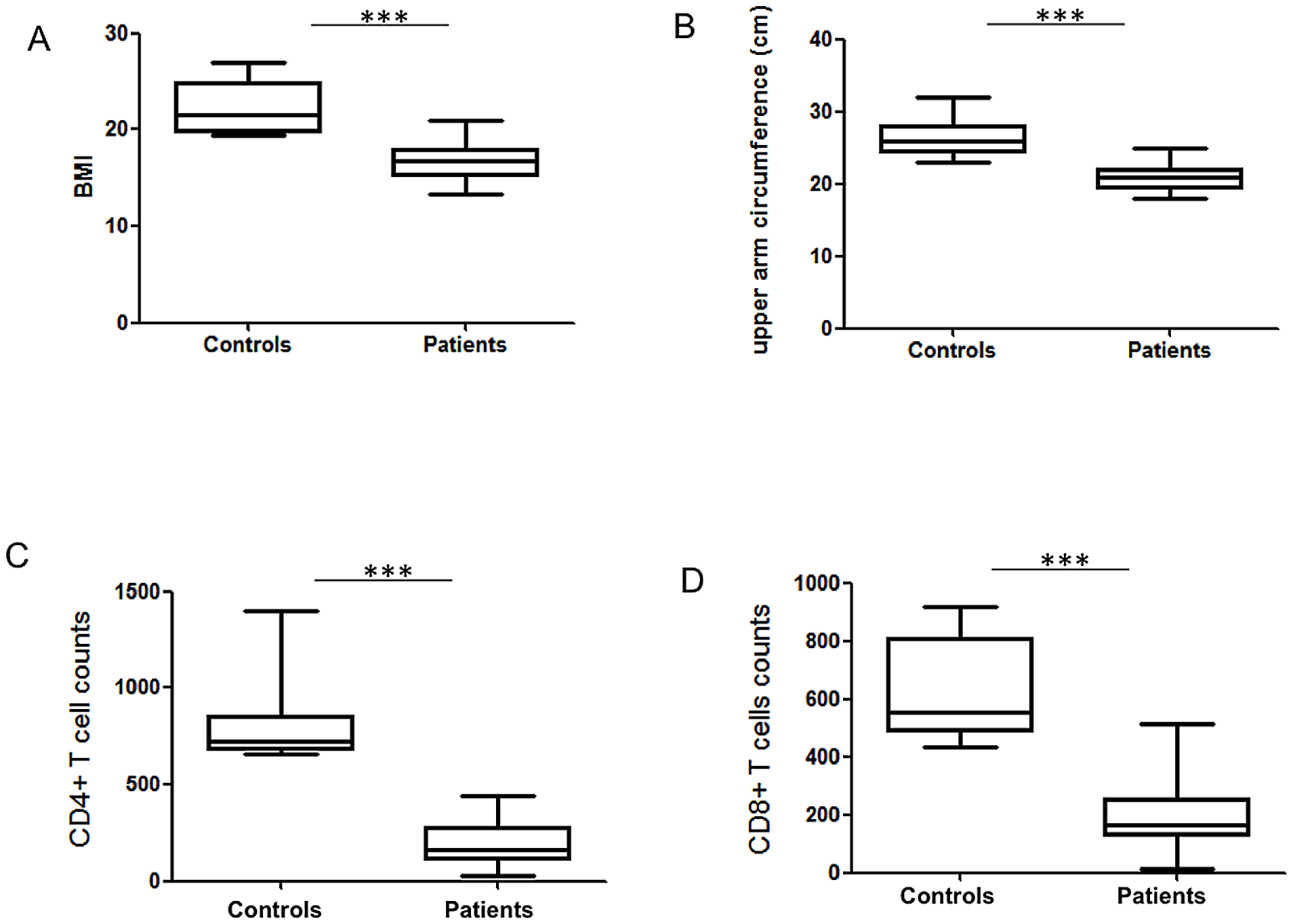
BMI, upper arm circumference and CD4^+^ and CD8^+^ T cell counts. BMI (A) and circumference of the upper arm (B) were compared in controls (n = 12) and patients (n = 25). CD4^+^ (C) and CD8^+^ T (D) cell counts in the blood of controls (n = 10) and patients (n = 25) were measured by flow cytometry. Ethiopian national reference range for CD4^+^ T cells counts (cells/µl of blood) = 500–1300; Ethiopian national reference range for CD8^+^ T cells counts (cells/µl of blood) = 320–1800. Box = interquartile range and median; whiskers = range. Patients = VL patients before treatment.

All patients recruited in this study were male with a median age of 22±1.1 years and presented with severe VL as shown by the clinical data presented in Table 1: all but one patients had enlarged spleen and/or liver and parasites were detected in the spleen or bone marrow aspirates of all patients. As shown in Table 1, the duration of illness varied from 2 to 24 weeks. In addition, the following parameters were measured: platelet and white blood cell counts, hematocrit and haemoglobin. Platelets and WBC counts were noticeably lower than the normal range in the large majority of the patients (Table 2). In addition, all but one patient had hematocrit and haemoglobin levels below the lower limit of normal (Table 2). The nutritional status of VL patients was determined by calculating their body mass index (BMI) and their upper arm circumference: the majority of the patients had a BMI below 18.5 (median±SEM: 16.5±0.3): out of 25 patients, 11 were malnourished [22] (BMI<18.5) and 12 were severely malnourished [22] (BMI<16); the BMI of all controls were above 18.5 (21.5±0.8) (Figure 1A). We did not find a significant difference between the BMI of the 4 endemic controls and the 8 controls recruited among the staff of the hospital (20.7±1.2 vs 22.4±0.7 respectively, p = 0.2828, data not shown). The median arm circumference was also significantly lower in VL patients (controls: 26.0±0.4 cm, patients: 21.0±0.8 cm, *p*<0.0001, Figure 1B). CD4^+^ and CD8^+^ T cell counts were assessed and results in Figures 1C and D show that they were markedly reduced in the blood of VL patients (CD4^+^ T cell counts (cell/µl blood) = 159.0±22.7; CD8^+^ T cell counts: 164.0±24.7). CD4^+^ and CD8^+^ T cell counts from controls were in the reference range (553.5±53.9 and 724.4±70.1 cells/µl blood, respectively).

**Table 1:** Clinical data.

Patient (all male)	Age (years)	Spleen size (cm)	Liver size (cm)	Parasite load (Spleen)	Parasite load (Bone marrow)	Duration of Illness (weeks)
1	20	3	3		1+	4
2	23	5	Not palpable		1+	16
3	18	17	4	4+		24
4	24	5	3		3+	8
5	16	Not done	Not done	4+		12
6	30	7	3	3+		4
7	20	12	3		1+	24
8	28	11.5	Not palpable	3+		4
9	25	8	Not palpable	2+		4
10	20	5	Not palpable	3+		2
11	18	15	4	4+		4
12	25	7	3		2+	16
13	18	12	5	4+		12
14	38	11	4	5+		12
15	24	6	3.5		2+	12
16	19	8	Not palpable	4+		3
17	22	5	Not palpable	4+		4
18	20	5	Not palpable	1+		4
19	32	4	5		3+	8
20	22	13	6	3+		12
21	18	Not palpable	Not palpable		1+	4
22	25	10	4		3+	8
23	20	7	5	5+		4
24	27	3	4		2+	8
25	16	12	3	4+		4
26	25	8	4	1+		4

Spleen and liver size = measurement below left costal margin and right subcostal margin (respectively).

**Table 2:** Haematological data.

Patients	Platelets(×10^3^)	White blood cells (×10^3^)	Hct (%)	Hb (g/dl)
1	190	4.6	38.4	12.1
2	14	1.2	25.9	7.8
3	134	1.9	24.7	7.4
4	38	0.8	23.8	7.2
5	Not done	Not done	Not done	Not done
6	78	0.9	24.1	7.3
7	19	2.3	17.1	5.3
8	40	1	25.3	7.7
9	45	1.8	29.8	9.4
10	125	2.7	29.2	8.3
11	45	1.4	28.9	9
12	34	1	11.9	3.9
13	93	1.3	21.9	7
14	75	1.5	25.2	8.2
15	46	1.3	23.7	7
16	73	1.9	21.1	6.9
17	133	2.2	23	7.2
18	87	3	29	9.4
19	65	1.9	21.4	6.59
20	126	2	22.1	6.6
21	43	2	12.6	3.9
22	27	1.1	26.1	8
23	43	2.5	21.1	6.3
24	57	1	25.3	7.9
25	67	2.4	24	7
26	49	1.6	21.1	Not obtained
**Median**	**57±8.6**	**1.8±0.2**	**24.0±1.1**	**7.3±0.3**

Hct = hematocrit; Hb = haemoglobin.

Normal range: platelets (×10^3^) = 150–450; white blood cells (×10^3^) = 4.5–10.5; Hct (%) = 35–60; hb (g/dl) = 11–18.

The white blood cells were counted in total blood before Ficoll using a COULTER Ac•T diff Hematology Analyzer and are expressed as number of WBC (×10^3^) per µl of blood.

### Arginase activity is higher in PBMCs of VL patients

**Figure 2:**
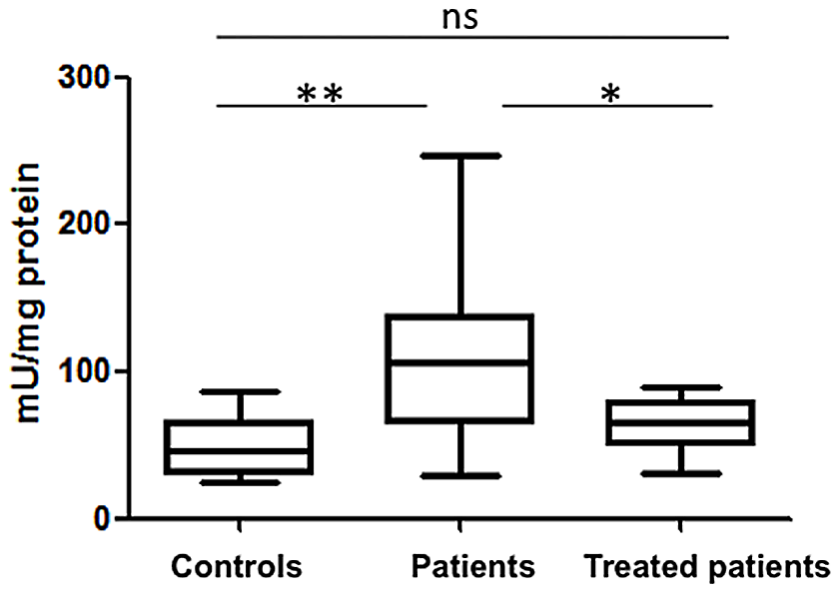
Arginase activity in PBMCs. PBMCs were isolated by density gradient from the blood of controls (n = 10), VL patients (n = 14) and treated VL patients (n = 12) and the activity of arginase was measured by enzymatic assay. Box = interquartile range and median; whiskers = range. Statistical significance was determined by a two-tailed Mann-Whitney test. NS = not significant. Patients = VL patients before treatment; treated patients = VL patients after 3–4 weeks treatment.

As shown in Figure 2, statistically significantly higher levels of arginase activity were measured in PBMCs of VL patients as compared to controls (patients: 106.6±11.0, controls: 45.9±6.7 mU/mg protein respectively, *p* = 0.0045). Following 3–4 weeks of treatment, these levels were considerably lower (treated patients: 64.8±5.3 mU/mg protein, *p* = 0.0108). These results show that the active phase of visceral leishmaniasis coincides with higher levels of arginase activity in PBMCs.

### CD15^+^ Arginase^+^ cells are more frequent among PBMCs of VL patients

**Figure 3:**
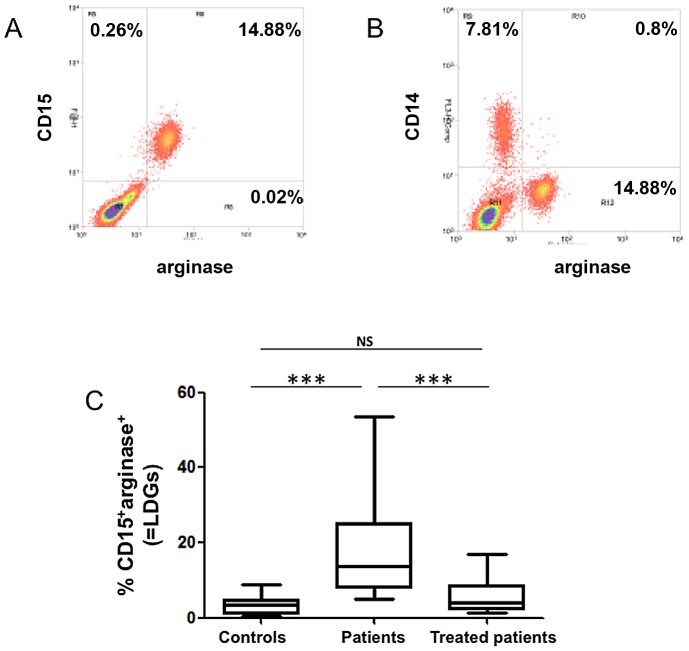
Phenotype and frequency of arginase-expressing cells in PBMCs. PBMCs were isolated by density gradient from the blood of controls (n = 14), VL patients (n = 17) and treated VL patients (n = 12). The phenotype of arginase-expressing cells was determined by flow cytometry. (A) Dot plot profile of CD15^+^ arginase^+^ cells, data show the results of one representative experiment out of 17 independent experiments; (B) dot plot profile of CD14^+^ arginase^+^ cells, data show the results of one representative experiment out of 17 independent experiments. (C) Percentages of CD15^+^arginase^+^ cells in the PBMCs of controls (n = 14), VL patients (n = 17) and treated VL patients (n = 12). Box  =  interquartile range and median; whiskers = range. Statistical significance was determined by a two-tailed Mann-Whitney test. NS = not significant. Patients = VL patients before treatment; treated patients = VL patients after 3–4 weeks treatment.

To determine the phenotype of arginase-producing cells, a combination of extracellular (anti-CD14 and anti-CD15) and intracellular (anti-arginase) markers was used [19]. The large majority of arginase-expressing cells in PBMCs of patients express CD15, a typical marker of neutrophils (14.9% of 15.1% = 98.3%, Figure 3A); the phenotype of arginase-expressing cells was similar (CD14^−^CD15^+^arginase^+^) in the PBMCs of controls and treated patients (data not illustrated). The frequency of CD15^+^ cells expressing arginase was >93.1% in all 17 patients and controls tested (data not illustrated). Following density gradient purification, neutrophils co-purify in the erythrocyte fraction and not with the PBMCs. However, here we identify arginase-expressing CD15^+^ cells in the PBMC fraction, this population will therefore be referred to as low-density granulocytes (LDGs). The frequency of LDGs was statistically significantly higher in the PBMCs of VL patients as compared to controls (patients: 13.6±3.4, controls: 3.5±0.7, *p* = 0.0001, Figure 3C). Following 3–4 weeks of treatment, the frequency of LDGs was considerably lower (treated patients: 4.1±1.3, *p* = 0.0006). Similarly, the number of LDGs per ml of blood was higher in patients as compared to controls and treated patients (Table 3).

**Table 3:** Absolute numbers of LDGs, CD14^+^ cells and white blood cells.

	Controls	Patients	Treated patients
**LDGs/ml blood**	18.8±6.9×10^3^	28.7±7.6×10^3^	14.3±5.3×10^3^
**CD14^+^ cells/ml blood**	8.3±1.3×10^4^	1.3±0.7×10^4^	5.2±1.1×10^4^
**White cells/ml blood**	6.0±0.8×10^5^	1.7±0.5×10^5^	3.5±0.3×10^5^

The white blood cell counts were determined after Ficoll using trypan blue exclusion and are expressed as cell number per ml of blood.

**Figure 4:**
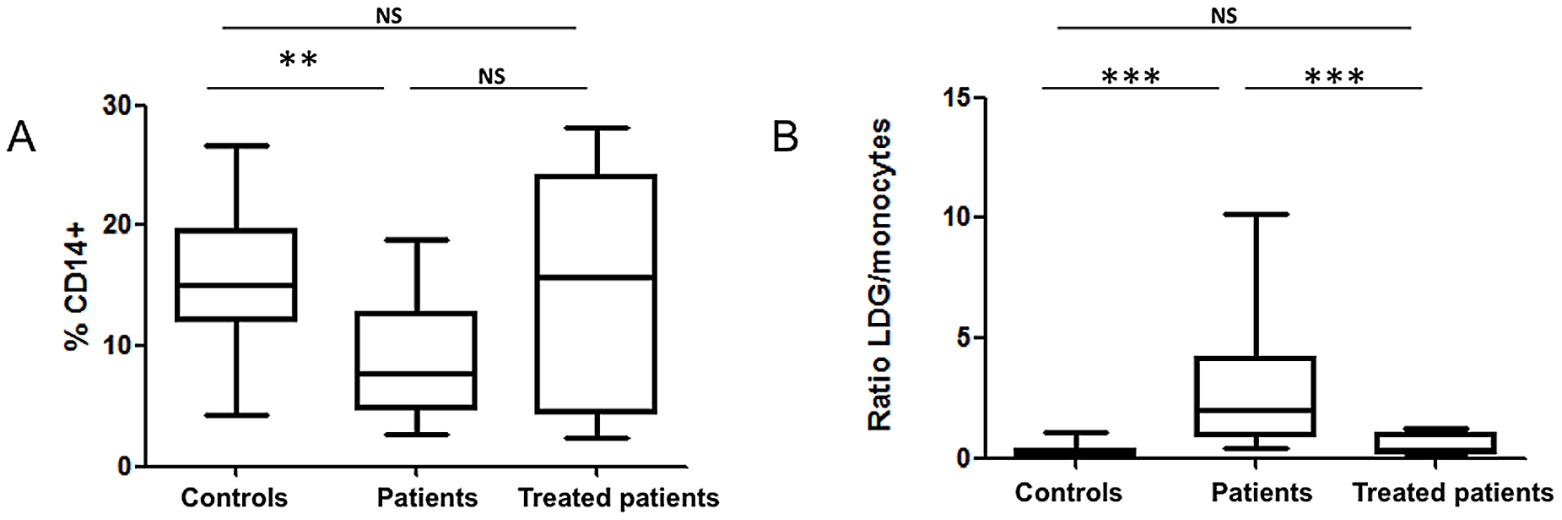
Frequency of LDGs and monocytes in PBMCs. PBMCs were isolated by density gradient from the blood of controls (n = 14), VL patients (n = 17) and treated VL patients (n = 12), (A) the frequencies of monocytes were determined by flow cytometry and (B) the ratio of low-density granulocytes (LDGs) to monocytes was calculated by dividing the percentages of LDGs by the percentages of monocytes. Box = interquartile range and median; whiskers = range. Statistical significance was determined by a two-tailed Mann-Whitney test. NS = not significant. Patients = VL patients before treatment; treated patients = VL patients after 3–4 weeks treatment.

CD14^+^ cells of the large majority of patients (14 out of 17) did not express arginase (the percentages of CD14^+^ arginase^+^ cells were <1%, Figure 3B), with only a small percentage of CD14^+^arginase^+^ cells detected in the PBMCs of 3 patients (1.7%, 3.5% and 2.8%, data not illustrated). The percentages of CD14^+^ cells were lower during the active phase of the disease (controls: 15.1±1.5 and patients: 7.8±1.2, *p* = 0.0040), and did not change significantly after 3–4 weeks of treatment (patients: 7.8±1.2 and treated patients: 15.7±2.7, *p* = 0.1013)(Figure 4A). Similar results were obtained with the number of CD14^+^ cells per ml of blood (Table 3). The ratio of LDGs versus monocytes was higher before treatment in VL patients (1.97±0.63) as compared to patients after 3–4 weeks of treatment (0.34±0.12, *p* = 0.0004) and controls (0.19±0.09, *p*<0.0001)(Figure 4B). Of note, the white blood cell count per ml of blood was lower in patients before the treatment as compared to controls and to treated patients (Table 3).

### Increased degranulation of LDGs in PBMCs of VL patients

**Figure 5:**
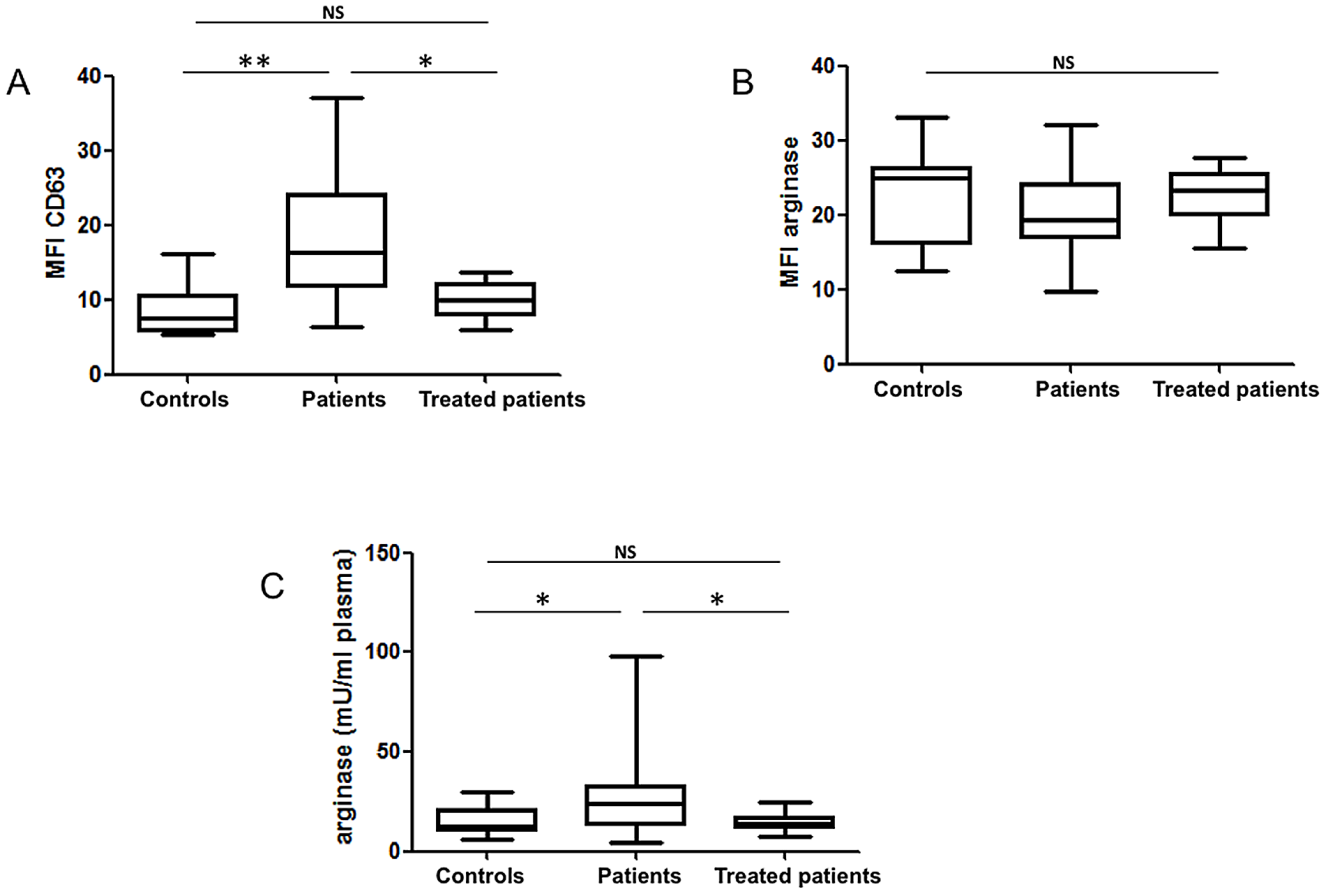
Phenotype of arginase expressing LDGs and arginase plasma levels. PBMCs were isolated by density gradient from the blood of controls [n = 8 (A) and n = 13 (B)], VL patients [n = 10 (A) and n = 16 (B)] and treated VL patients [n = 8 (A) and n = 12 (B)] and the mean fluorescence intensity of CD63 (A) and arginase (B) in CD15^+^ cells was determined by flow cytometry. (C) Plasma was isolated by centrifugation of the blood of controls (n = 21), VL patients (n = 21) and treated VL patients (n = 14) and the activity of arginase was measured by enzymatic assay. Box = interquartile range and median; whiskers = range. Statistical significance was determined by a two-tailed Mann-Whitney test. NS = not significant. Patients = VL patients before treatment; treated patients = VL patients after 3–4 weeks treatment.

Activation of neutrophils is accompanied by exocytosis of arginase-containing azurophilic granules [23], which express CD63. Release of these granules results in the incorporation of CD63 into the membrane of neutrophils [24]. Therefore, we measured the expression levels of CD63 and arginase in LDGs. As shown in Figure 5A, CD63 MFI was statistically significantly higher on LDGs isolated from the PBMCs of patients before treatment as compared to controls (patients: 16.4±3.1, controls: 7.5±1.3, *p* = 0.0066) and to treated patients (10.0±0.9, *p* = 0.0232), suggesting that the levels of degranulation are higher in LDGs from patients before treatment. Since arginase has been shown to be contained in the azurophilic granules found in the cytoplasm of neutrophils, we assessed its MFI in LDGs: the lower median values observed in the LDGs of VL patients did not reach statistical significance (controls: 24.9±1.7 vs VL patients: 19.4±1.4, *p* = 0.3126; VL patients: 19.4±1.4 vs treated patients: 23.4±1.0, *p* = 0.0855). Next, we measured the levels of arginase activity in the plasma and our results show that arginase activities were statistically significantly higher in the plasma of patients before treatment (23.9±4.8 mU/ml plasma) as compared to controls (12.8±1.4 mU/ml plasma, *p* = 0.0117) and treated patients (13.9±1.1 mU/ml plasma, *p* = 0.0168)(Figure 5C).

### Lower levels of L-arginine in the plasma coincide with downregulation of CD3ζ in CD4^+^ T cells

**Figure 6:**
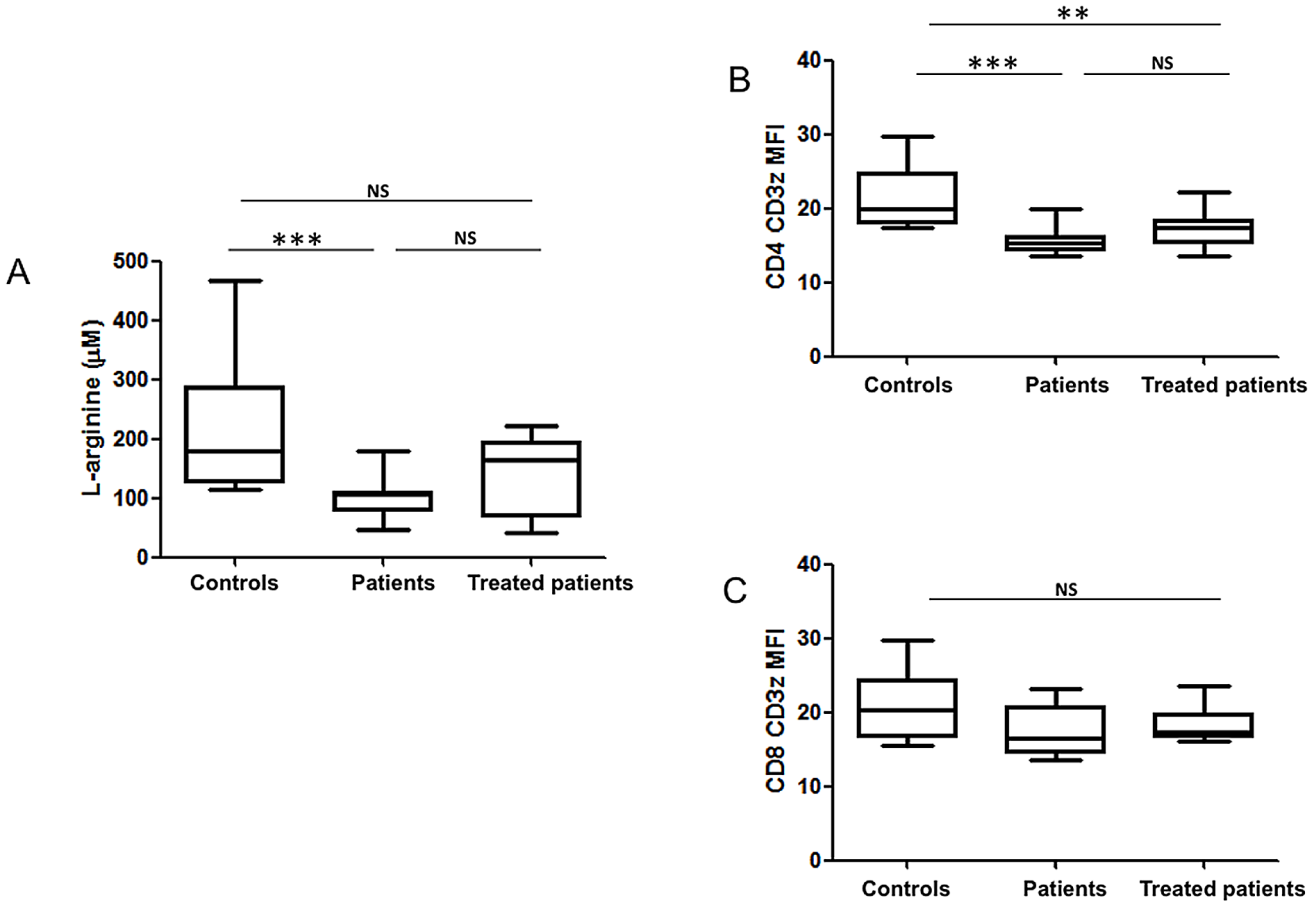
L-arginine levels and CD3ζ expression in CD4^+^ and CD8^+^ T cells. Plasma was isolated by centrifugation of the blood of controls (n = 11), VL patients (n = 11) and treated VL patients (n = 11) and (A) the levels of L-arginine were measured by HPLC. PBMCs were isolated by density gradient from the blood of controls (n = 10), VL patients (n = 10) and treated VL patients (n = 10) and the mean fluorescence intensity of CD3ζ was determined in CD4^+^ T cells (B) and CD8^+^ T cells (C). Box = interquartile range and median; whiskers = range. Statistical significance was determined by a two-tailed Mann-Whitney test. NS = not significant. Patients = VL patients before treatment; treated patients = VL patients after 3–4 weeks treatment.

Since the results shown in Figure 5C show higher levels of arginase activity in plasma from VL patients, we measured the levels of L-arginine in the plasma. As shown in Figure 6A, a sharp reduction in L-arginine levels was observed in the plasma of patients before treatment (105.6±12.4 µM) as compared to healthy controls (179.0±31.6 µM, *p* = 0.0009); the increased median levels of L-arginine did not reach statistical significance in the plasma of patients after 3–4 weeks of treatment (164.7±19.8 µM, *p* = 0.2934). It has been shown that decreased levels of L-arginine result in T cell suppression, as measured by decreased expression of CD3ζ [17], [19]. We next assessed the expression levels of CD3ζ in CD4^+^ and CD8^+^ T cells in PBMCs: statistically significantly lower CD3ζ MFI in CD4^+^ T cells from patients before treatment (15.4±0.6) was observed as compared to controls (20.0±1.3, *p* = 0.0008); higher CD3ζ MFI in CD4^+^ T cells that shows a trend towards significance was observed in the treated patients (17.4±0.8, *p* = 0.081)(Figure 6B). No statistical significance was observed in CD3ζ MFI in CD8^+^ T cells (patients: 16.5±1.1, controls: 20.4±1.5, treated patients: 17.4±0.8, Figure 6C).

## Discussion

PBMCs from patients with active VL lose the ability to mount an antigen-specific immune response, as shown by the inability of their T cells to proliferate and produce IFN-γ; this is however reversed after successful chemotherapy (summarised in [12], [13]). Here we identified a novel mechanism in human visceral leishmaniasis that might account for the poor T cell responses associated with active visceral leishmaniasis. We show that the activity of arginase, an enzyme that has been associated with immunosuppression, is higher before treatment in PBMCs and plasma of VL patients and decreases rapidly following successful treatment. Indeed, our results show that as early as 3 weeks post treatment, the levels of arginase are similar to those of controls. Similarly, the frequency of arginase-expressing cells in the PBMCs and their absolute counts were shown to be considerably greater during active disease, indicating that increased arginase activity and increased frequency of arginase-expressing cells are hallmarks of active visceral leishmaniasis. Further, we show that increased arginase activity and frequency and absolute numbers of LDGs in the blood of patients with active VL coincide with lower levels of L-arginine in the plasma and lower expression levels of CD3ζ in CD4^+^ T cells. We have previously shown that increased levels of arginase activity correlated with disease severity in HIV seropositive patients [21]. Here, no significant correlation was observed between any of the clinical and haematological parameters or the nutritional status (data not shown) and arginase activity or frequency of LDGs. This is likely to be due to the fact that the patients recruited in our study all presented with severe VL. Therefore, our results suggest that arginase activity is significantly increased in VL patients presenting with severe manifestations of the disease.

We and others have already described LDGs [19], [21], [25], [26]; following density gradient centrifugation, these cells co-purify with PBMCs and not with the erythrocyte fraction, suggesting that they are activated granulocytes [27], which have changed their density. The degree of activation of neutrophils and degranulation depends on the strength of the activating signal and increased activation results in the release of granules in the following order: 1) secretory granules; 2) gelatinous (tertiary) granules; 3) specific (secondary) granules and 4) azurophilic (primary) granules. CD63 is found in the membrane of azurophilic granules and we show here that the MFI of CD63, whose upregulation parallels release of azurophilic granule by activated neutrophils [24], [28], is significantly higher on LDGs from active VL patients. Whereas we have previously shown that arginase is contained in azurophilic granules [23], arginase has also been detected in gelatinous granules [29]. Since azurophilic granules are the last granules to be released and since CD63 is selectively upregulated on neutrophils following release of azurophilic granules [24], [28], in agreement with our previous results [23], we can conclude that arginase-containing azurophilic granules are released. And indeed, the levels of arginase activity are higher in the plasma from patients during the active phase of the disease. These results suggest that LDGs have degranulated and released their arginase. We have recently shown that a similar population of LDGs is significantly increased in the blood of HIV seropositive patients with low CD4^+^ T cell counts: this population also expressed higher CD63 and lower arginase MFIs; in addition, the levels of CD11b, CD15, CD33, CD66b, CD63 were significantly higher and that of CD16 significantly lower as compared to NDGs [30]. The majority of LDGs in HIV patients were mature segmented neutrophils [30]. However, the origins of these cells as well as the factors resulting in the degranulation of neutrophils during VL are still unclear. It is possible that during VL, neutrophils are activated in the spleen and in the liver and that a fraction of these activated neutrophils recirculate and are therefore detected in the PBMCs (Figure 3). An extensive and systemic activation of neutrophils would result in the release of large amount of arginase, widespread depletion of L-arginine and major disruption of many cellular and organ functions and is therefore unlikely.

In contrast to our results, a recent study by Mukhopadhyay et al showed that the levels of arginase in the plasma of VL patients in India are not affected by treatment with sodium antimony gluconate [31]. These discrepancies are likely to be due to the different techniques used to measure arginase activity: in the present study, we deducted the levels of urea present in the plasma from the values obtained following activation of arginase. Of note, even though our results suggest that LDGs have degranulated, we cannot conclude from the present study that the increased arginase we detect in the plasma of VL patients is coming from LDGs, as it could also have been released from damaged hepatocytes. The increased arginase activity in PBMCs and plasma coincides with lower levels of L-arginine. We have previously shown that increased arginase activity, a marker of disease severity in HIV+ patients, correlated with decreased levels of L-arginine [21]. Another study has also described a correlation between elevated arginase activities and lower levels of L-arginine in patients with pulmonary tuberculosis, that was reversed following successful treatment [32]. In our study, in addition to the increased arginase activity measured in the plasma of patients with active VL, it is possible that malnutrition also impacted on the lower levels of L-arginine. Out of 25 patients, 11 were malnourished and 12 were severely malnourished. Since malnutrition results in reduced levels of L-arginine in the plasma [33], [34], [35], it is likely that this has also contributed to the lower levels of L-arginine in the plasma. Further, since the patients do not get nutritional supplements during treatment, malnutrition could also explain why the levels of L-arginine do not increase after 3–4 weeks of treatment. We had a limited number of patients in this study and our cohort of controls consisted of 4 endemic controls and 10 members of the staff of the hospital; we did not find significant differences between the nutritional status of these 2 subgroups of control. It will be interesting to compare the levels of L-arginine in the plasma of a large cohort of controls coming from endemic areas and from patients with active VL, to estimate the contribution of increased arginase and/or protein-energy malnutrition to the lower levels of L-arginine.

It has been shown previously that arginase-induced L-arginine depletion results in impaired T cell responses (reviewed in [14], [15], [16], [36]. Our results suggest that during active VL, LDGs release arginase and we speculate that arginase-induced L-arginine depletion contributes to the poor antigen-specific T cell response that is an immunological hallmark of active VL; it is also possible that this mechanism negatively impacts on the capacity of immune cells to respond to other antigens and might therefore account for the frequent opportunistic infections observed in VL patients.

The impaired *Leishmania*-specific T cell responses observed during active VL is reversed in cured patients ([6] and reviewed in [12], [13]). Our results show that CD3ζ MFI are significantly lower in CD4^+^ T cells, but not in CD8^+^ T cells, and 3–4 weeks post treatment, the increased median in CD3ζ in both cell type were not significantly different. It is possible that this lack of significance in CD3ζ in CD8^+^ T cells might be due to the limited number of patients recruited in this study. We have previously shown that CD4^+^ and CD8^+^ T cells both display decreased CD3ζ expression levels and this coincides with increased arginase activity [37]. The differences in L-arginine levels in the plasma were not statistically significant either; it is possible that this is due to the fact that the blood was collected only after 3–4 weeks of treatment.

The observation that PBMCs from patients with active VL mount a poor antigen-specific response is in apparent contradiction with recent studies, which show a clear production of IFN-γ using a whole blood assay [38], [39]: the authors of these studies have suggested that during the density gradient centrifugation used to isolate PBMCs, factors important for the production of IFN-γ might be removed. Our results obtained in a separate study show that heparin has a remarkable effect on the survival of LDGs. Indeed, our results show a dramatic decrease in the frequencies of LDGs when the blood was collected into sodium heparin as compared to EDTA: we compared the frequency of CD15^+^ arginase^+^ cells in the blood of 5 patients, collected in sodium heparin or EDTA and observed an average of 75.56±17.26% less CD15^+^arginase^+^ cells in blood collected in heparin compared to EDTA (% of LDGs in blood collected on EDTA = 2.48±0.96 vs 0.59±0.55 for blood collected with heparin (n = 5), *p*>0.05) [40]. Since in the above-mentioned studies [38], [39], blood was collected into heparin, it is possible that the resulting low frequency of LDGs was not sufficient to suppress the production of IFN-γ. Notably, neutrophils are extremely difficult to freeze and indeed, we were not able to freeze LDGs: we froze the PBMCs of 8 patients in FCS containing 10% DMSO, and following careful thawing of the cells, we observe an average reduction of 97.3±4.9% in the frequency of CD15^+^arginase^+^ cells. Similar results were recently published [41], where it was shown that the frequency of CD15^+^ cells in PBMCs was significantly reduced after cryopreservation. Since we did not have the facilities to perform stimulation assays with PBMCs from VL patients and since it is not possible to cryopreserve LDGs to send them in another laboratory with adequate facilities, we were therefore not able to directly demonstrate that LDGs have the ability to suppress T cell responses.

We have shown in an experimental model of cutaneous leishmaniasis that increased arginase activity is highest at the site of pathology, and significantly lower in the draining lymph nodes [17]. Further, we have recently performed a study with patients with localised cutaneous leishmaniasis and have shown that consistent with the mouse model, arginase activity is increased in the lesions, but not in the periphery [37]. Therefore, it is possible that the higher frequencies of LDGs we observed in the PBMCs of VL patients before treatment are only a weak reflection of the events occurring at the local sites of *Leishmania* replication, such as spleen, liver or lymph nodes; and that considerably stronger T cell suppression is likely to occur in these sites. Indeed, a recent study showed in a mouse model of prostate-specific inflammation, that a population of Gr-1^+^ CD11b^+^ arginase^+^ cells isolated from the site of inflammation are more suppressive than those isolated from the periphery [42]. Further studies will be essential in assessing the strength of LDG-mediated T cell suppression in different compartments and to show whether arginase-mediated L-arginine metabolism is a key element in the outcome of VL.

We and others have shown that *Leishmania* parasites express their own arginase [43], [44], [45]. However, it is highly unlikely that parasite arginase accounts for the arginase we detect in the PBMCs and in the plasma as it is very difficult to detect parasite in the blood of VL patients [46].

In summary, here we show that high arginase activity in PBMCs is a marker of active visceral leishmaniasis and we propose that arginase-induced L-arginine depletion contributes to disease severity in these patients.
